# Non-respiratory health risks and mortality associated with fighting bushfires (wildfires): a systematic review

**DOI:** 10.1007/s00420-025-02138-7

**Published:** 2025-04-09

**Authors:** Asmare Gelaw, Win Wah, Deborah C. Glass, Malcolm R. Sim, Ryan Hoy, Janneke Berecki-Gisolf, Karen Walker-Bone

**Affiliations:** 1 Monash Centre for Occupational and Environmental Health, School of Public Health and Preventive Medicine, Faculty of Medicine, Nursing and Health Sciences, Monash University, 553 St Kilda Road, Melbourne, VIC 3004 Australia; 2 Department of Respiratory Medicine, Alfred Health, Melbourne, Australia; 3 Monash Accident Research Centre, Monash University, Melbourne, Australia

**Keywords:** Systematic review, Wildfire, Bushfire, Firefighters, Mortality, Injury, Musculoskeletal injury, Morbidity, Health outcomes, Mental health

## Abstract

**Background:**

Bushfires (also known as wildland or forest fires) expose emergency responders to occupational hazards under exceptional circumstances. Whilst the health impacts of structural firefighting have been studied, less is known about the non-respiratory health impacts or risk of mortality amongst bush firefighters, who can be volunteers. More information about health risks is needed to generate effective prevention strategies.

**Objective:**

To critically evaluate and synthesise the published evidence about the non-respiratory health risks and risk of mortality associated with bushfire fighting.

**Methods:**

A systematic literature search was conducted in Medline, Scopus, and Embase to identify studies evaluating morbidity or mortality or associated risk factors among bushfire fighters. The quality of included studies was evaluated twice independently using a specific quality assessment tool.

**Results:**

Twenty-seven studies were included. 11(41%) were assessed as moderate quality and 16(59%) as low quality. There is a growing body of evidence for adverse short-term impacts of bushfire fighting on mental health and injuries. Linkage studies showed that volunteer firefighters had lower mortality and cancer risk in their late forties compared to the general population.

**Conclusion:**

Most studies relied on cross-sectional and retrospective designs without comparison groups, limiting the ability to draw robust conclusions. It is essential to conduct higher-quality research using prospective designs and longer-term follow-up to better understand the health outcomes of bushfire fighting, particularly given the anticipated increase in the frequency and severity of bushfires.

**Supplementary Information:**

The online version contains supplementary material available at 10.1007/s00420-025-02138-7.

## Introduction

The frequency and intensity of bushfires (also known as wildfires or forest fires) have been increasing, attributable to global warming and climate change (Shivanna 2022; D’Evelyn et al. 2022). In the past decade, widespread severe bushfires have occurred globally, including in Australia, Europe, and the Americas (Senande-Rivera et al. 2022; Australian Institute for Disaster Resilience 2014, 2020; European Environment Agency., 2024; Zong et al. 2022). The severity has caused damage to human life, property, public health, and ecosystems (Australian Institute for Disaster Resilience 2014, 2020; European Environment Agency., 2024; Zong et al. 2022; Deb et al. 2020). Bushfires generally occur in rural areas with sparse populations, and therefore, the responding workforce includes paid and volunteer firefighters, often from a wide range of occupational backgrounds (for example, in Australia, staff employed by the parks and wildlife authorities are mobilised as bushfire fighters). Moreover, the exposure associated with bushfires can be similar to, and different from, those associated with structural firefighting, including intense heat; risk of burns from the fires and embers; traumatic injury; musculoskeletal stressors; high levels of small Particulate Matter (PM2.5) and other pollutants, long working hours under stressful conditions and shift/night work, rest periods and temporary accommodation near to the fire site and away from home, all of which can increase the risk of health consequences (Finlay et al. 2012; Ademi et al. 2023; Duckett et al. 2020).

Although some health impacts of exposure to bushfires have been described, (Rodney et al. 2021; Liu et al. 2015; Jegasothy et al. 2023) including negative effects on respiratory (Ademi et al. 2023), immune (Hamon et al. 2018) and cardiovascular systems (Reisen and Brown 2006; Dennekamp et al. 2015) most research has been amongst the general population resident local to the fire.(Rodney et al. 2021; Liu et al. 2015; Jegasothy et al. 2023; Hamon et al. 2018; Reisen and Brown 2006). It could be expected that health effects amongst those actively involved in firefighting will be more extreme, given that they can be exposed intensely for long periods and possibly on multiple occasions. They may also witness events at fire sites, which increase the risk of psychological consequences.

While the respiratory risks of bushfire fighting have been explored, (Wah et al. 2025) there is a limited synthesis of evidence regarding other non-respiratory health conditions and mortality outcomes. Therefore, the purpose of this systematic review was to synthesise existing evidence about the longer-term non-respiratory health impacts of bushfire fighting, including the risk of mortality and identifying factors associated with adverse health impacts. By better understanding the harm to health, we can start to develop strategies for prevention. Additionally, the aim was to identify any research gaps, including the absence of relevant research or lack of good-quality studies.

## Methods

### Design and protocol registration

This systematic review was conducted and reported in compliance with the Preferred Reporting Items for Systematic Reviews and Meta-Analyses (PRISMA) guidelines (Shamseer et al. 2015). The systematic review protocol was registered with the International Prospective Register of Systematic Reviews (PROSPERO) on 20 July 2023 (Registration number: CRD42023443905).

### Search strategy

The following databases were searched up to January 7, 2025: Medline, Scopus, and EMBASE. The search used a comprehensive range of relevant terms to ensure the inclusion of all potentially eligible results about morbidities and mortality among first responders, whether paid or unpaid, who actively engaged in bushfire fighting (the full search strategy is included in Appendix 1 as supplementary material). The search was limited to peer-reviewed English-language articles reporting research involving humans and bushfires (“wild(land) fires” and “forest fires” also searched). No restrictions were placed on the date of publication. Additional articles were identified by manually searching the reference lists of included articles and conducting forward citation searches.

### Eligibility criteria

Eligible studies were those which investigated the association between adults (paid or volunteer) who were involved in bushfire fighting and one or more of injury, morbidity, and mortality, excluding respiratory disease(s). Qualitative studies, grey literature, editorials, and conference abstracts were excluded. Only peer-reviewed, full-text articles in English were included. Studies focusing on structural firefighting, training, and/or bushfire preparation were excluded.

### Study selection

Search results were imported to Covidence (Innovation 2018). Two reviewers independently reviewed the titles, abstracts and full texts of identified studies based on the pre-specified eligibility criteria. Discrepancies were settled by consensus.

### Risk of bias assessment

Papers selected for inclusion were assessed for risk of bias using a specific quality assessment tool recommended for exploring the health impacts of occupational wildfire smoke exposure (Groot et al. 2019). The tool includes five domains: Exposure assessment (scores one to three), outcomes assessment (scores one to three), controlling for potential confounders (scores null or one), sample size (score null, one or two), and study design (score 0–4). The quality assessment was based on the total score: a score of ≥ 12 “high quality” ≥ 8 or ≤ 11 “moderate quality”, and ≤ 7 “low quality”. Two authors (AG and WW) scored each study independently, with the final rating agreed by consensus.

### Data extraction and synthesis

A data extraction tool was developed to retrieve the following information for each included study: authors’ last names, year of publication, country, sample size, study design, and demographic characteristics (age, sex) of the participants. Furthermore, information on exposure measures, outcomes (type and prevalence of health conditions), methodologies for assessing outcomes, intervals between bushfire fighting exposure and observed health outcomes, as well as effect sizes and major findings were extracted.

## Results

**Fig. 1:**
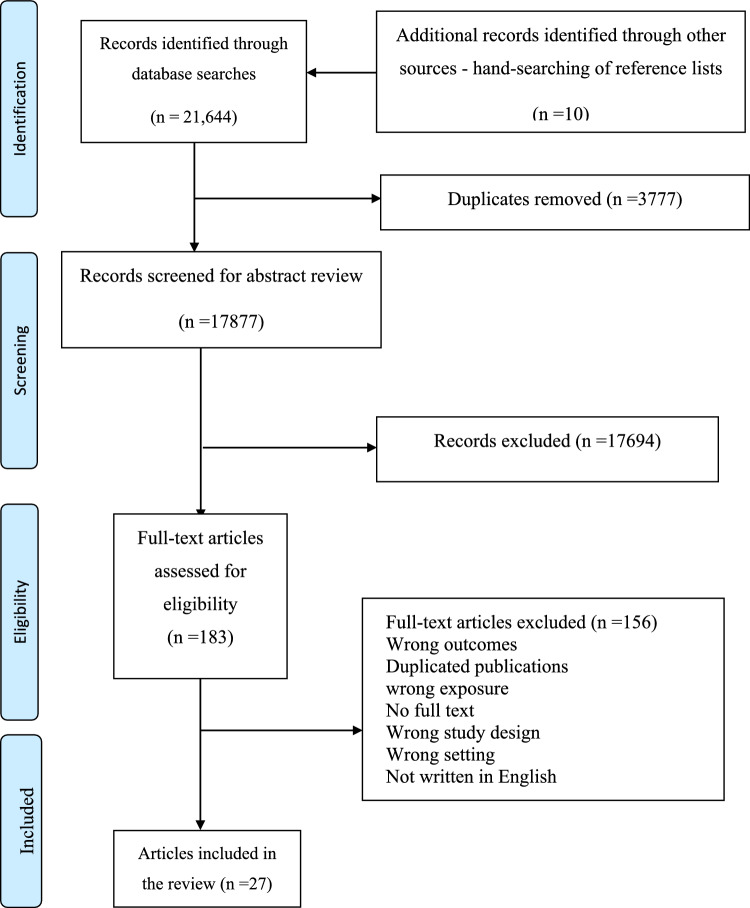
PRISMA flow diagram of the selection of studies

The database search yielded 21,654 potentially eligible papers, excluding 3777 duplicates. After de-duplication, 17,877 articles remained for title and abstract screenings. In total, 183 papers were screened at the full-text stage, amongst which 27 studies met the inclusion criteria (Fig. 1).

### Study characteristics

All studies were observational, including 18 cross-sectional studies and 7 retrospective cohort studies and two studies used cross-shift cohort design. Cross-shift cohort studies investigated changes in outcomes between shifts. Studies were retrieved from: the USA (n = 8) (Britton et al. 2013a, 2013b; Butler et al. 2017; Stanley et al. 2018; Jung et al. 2021, 2023; Hasan et al. 2023a, 2023b) Australia (n = 9)(McFarlane 1989; Spurrell and McFarlane 1993; Vincent et al. 2016; Doley et al. 2016; Glass et al. 2017, 2019; Berecki-Gisolf et al. 2024; Wah et al. 2024; Jaiswal et al. 2024), Canada (n = 4)(McGillis et al. 2017; Semmens et al. 2016; Moody et al. 2019; Jeklin et al. 2020), Israel (n = 2)(Amster et al. 2013; Leykin et al. 2013), Spain (n = 2) (García-Heras et al. 2022, 2024) and one each from Portugal,(Becker et al. 2023) and Greece (Psarros et al. 2018).

### Participants

The included studies ranged in number of participants from 20 (Stanley et al. 2018) to 102,073 (Glass et al. 2017) and, in total, involved over 300,000 individuals who had fought bushfires. Studies also evaluated the health of paramedics (Jung et al. 2021, 2023) and police officers (Amster et al. 2013). Three studies compared the health of bushfire fighters with community-based controls/general population (Hasan et al. 2023a; Glass et al. 2017) (Glass et al. 2019). Two studies compared the health of female firefighters with that of female nurses from a US cohort of Nurses’ Health Study II (Jung et al. 2021, 2023). Ten of the 24 studies included volunteer bushfire fighters (Butler et al. 2017; Jung et al. 2021, 2023; McFarlane 1989; Spurrell and McFarlane 1993; Vincent et al. 2016; Doley et al. 2016; Glass et al. 2019; Becker et al. 2023; Jaiswal et al. 2024) Five studies did not report the sex distribution(McFarlane 1989; Spurrell and McFarlane 1993; Britton et al. 2013a, 2013b; Butler et al. 2017) but, in those that did, the proportion of men ranged between 50(Stanley et al. 2018; Moody et al. 2019) and 100% (Glass et al. 2017; McGillis et al. 2017; Amster et al. 2013)(Leykin et al. 2013; Psarros et al. 2018). Three studies exclusively studied female bushfire fighters to explore impacts on reproductive health (Jung et al. 2021, 2023; Glass et al. 2019). Two studies explored workers’ compensation data (Berecki-Gisolf et al. 2024; Wah et al. 2024). The age of participants was not reported in five studies (Britton et al. 2013a, 2013b; Butler et al. 2017; Jung et al. 2021; Spurrell and McFarlane 1993) but, in the others, ranged from 24.6 (4.8) (Jeklin et al. 2020) to 42.2 (10.6) years (Doley et al. 2016).

### Exposure assessment measures

Exposure assessment varied by type and study. Some studies estimated exposure based on average hours spent firefighting (McFarlane 1989; Spurrell and McFarlane 1993), while others assessed the impact of multi-day firefighting deployments for specific incidents or shifts (Amster et al. 2013; Leykin et al. 2013). Some studies specifically measured exposure to wildfire smoke and/or chemical flame retardants (Leykin et al. 2013) whilst others drew on historical reports from agencies like the US Department of the Interior (Britton et al. 2013a; 2013b). In specific studies, the duration of exposure ranged from 15.6 h per specific incident to multi-day exposures, with some studies examining risk from long-term exposure over 10–20 years of service (Glass et al. 2017). None of the included studies reported about use of personal protective equipment during firefighting.

### Quality of included studies

The agreed quality assessment scores (Table 1) were an average of 7.2 with scores ranging from 4 to 11. Eleven were assessed as of moderate-quality (total score: 8–11) (McFarlane 1989; Britton et al. 2013a, 2013b; 2017, 2019; Jeklin et al. 2020; Becker et al. 2023; Hasan et al. 2023a, 2023b; Berecki-Gisolf et al. 2024; Wah et al. 2024) and the remaining 16 as low-quality (total score ≤ 7). (Spurrell and McFarlane 1993; Amster et al. 2013;  Leykin et al. 2013; Vincent et al. 2016; Doley et al. 2016; Semmens et al. 2016; Butler et al. 2017; McGillis et al. 2017; Stanley et al. 2018; Moody et al. 2019; Jung et al. 2021, 2023; García-Heras et al. 2022, 2024; Psarros et al. 2018; Jaiswal et al. 2024).

**Table 1:** Quality assessment of included studies of non-respiratory health risks and mortality amongst bushfire fighters

Authors	Exposure assessment • self-reported exposure severity = 1 • shift or season duration = 2 • individual- or area-level monitoring = 3	Outcome assessment • self-reported health outcomes = 1 • marker of acute physiological responses = 2 • objective measurement of diseases = 3	Control for potential confounders • no/unclear = 0 • yes = 1	Sample size in analysis • case report = 0 • < 50 participants = 1 • ≥ 50 participants = 2	Study design • case report or description = 0 • ecological or cross-sectional = 1 • case–control = 2 • cohort = 3 • randomized control trial = 4	Total scores	Quality level • high ≥ 12 • moderate 8–11 • low ≤ 7
(McFarlane 1989)	1	1	1	2	3	8	Moderate
(Spurrell and McFarlane 1993)	1	1	0	2	3	7	Low
(Britton et al. 2013a)	1	3	1	2	1	8	Moderate
(Britton et al. 2013b)	1	3	1	2	1	8	Moderate
(Amster et al. 2013)	1	1	0	2	1	5	Low
(Leykin et al. 2013)	1	1	0	2	1	5	Low
(Vincent et al. 2016)	2	1	0	1	3	7	Low
(Doley et al. 2016)	1	1	1	1	3	7	Low
(Semmens et al. 2016)	1	1	1	2	1	6	Low
(Butler et al. 2017)	1	3	0	1	0	5	Low
(Glass et al. 2017)	2	3	1	2	3	11	Moderate
(McGillis et al. 2017)	1	1	0	1	1	4	Low
(Stanley et al. 2018)	1	1	1	2	1	6	Low
(Glass et al. 2019)	2	3	1	2	3	11	Moderate
(Moody et al. 2019)	1	1	0	2	1	5	Low
(Jeklin et al. 2020)	2	2	1	1	3	9	Moderate
(Jung et al. 2021)	1	1	1	2	1	6	Low
(García-Heras et al. 2022)	1	1	1	2	1	6	Low
(Jung et al. 2023)	1	1	1	2	1	6	Low
(Psarros et al. 2018)	1	3	1	1	1	7	Low
(Becker et al. 2023)	3	3	1	2	1	10	Moderate
(Hasan et al. 2023a)	2	3	1	2	1	9	Moderate
(Hasan et al. 2023b)	2	3	1	2	1	9	Moderate
(Berecki-Gisolf et al. 2024)	2	3	1	2	1	9	Moderate
(Wah et al. 2024)	2	3	1	2	1	9	Moderate
(Jaiswal et al. 2024)	1	1	1	2	1	6	Low
(García-Heras et al. 2024)	2	1	0	2	1	6	Low

Thirteen of the low-quality studies were cross-sectional, including a wide range of methods for exposure assessment, outcome measurement, and methods to consider confounding (Amster et al. 2013; Leykin et al. 2013; Semmens et al. 2016; Butler et al. 2017; McGillis et al. 2017; Stanley et al. 2018; Moody et al. 2019; Jung et al. 2021, 2023; García-Heras et al. 2022, 2024; Psarros et al. 2018; Jaiswal et al. 2024).

Seventeen studies relied predominantly on self-reported exposure (McFarlane 1989; Spurrell and McFarlane 1993; Britton et al. 2013a, 2013b; Amster et al. 2013; Leykin et al. 2013; Doley et al. 2016; Semmens et al. 2016; Butler et al. 2017; McGillis et al. 2017; Moody et al. 2019; Jung et al. 2021, 2023; García-Heras et al. 2022; Psarros et al. 2018; Jaiswal et al. 2024) Likewise, 15 relied on self-reported health outcomes. (McFarlane 1989; Spurrell and McFarlane 1993; Amster et al. 2013; Leykin et al. 2013; Vincent et al. 2016; Doley et al. 2016; Semmens et al. 2016; McGillis et al. 2017; Stanley et al. 2018; Moody et al. 2019; Jung et al. 2021, 2023; García-Heras et al. 2022, 2024; Jaiswal et al. 2024) In eight studies, it was unclear whether there was control for confounding such as age, gender, baseline health status, or other occupational exposures. (Spurrell and McFarlane 1993; Amster et al. 2013; Leykin et al. 2013; Vincent et al. 2016; Butler et al. 2017; McGillis et al. 2017; Moody et al. 2019; García-Heras et al. 2024) Six studies had sample sizes below 50 participants.(Vincent et al. 2016; Doley et al. 2016; Butler et al. 2017; McGillis et al. 2017; Jeklin et al. 2020; Psarros et al. 2018).

### Health measures

The results of all included studies are shown in Table 2. The following non-respiratory health conditions were studied: mental health conditions, including suicide, injuries, arthritis and musculoskeletal surgery, cardiovascular diseases (CVD), arrhythmias, hypertension and hypercholesterolaemia, chronic pain, cancer, sleep and fatigue, reproductive health outcomes, and other health conditions. All-cause mortality was studied in two large retrospective cohort studies (Glass et al. 2017, 2019) and acute mortality from bushfires in the USA using surveillance data (Butler et al. 2017).

**Table 2:** Characteristics and results of the included studies which investigated non-respiratory health risks and mortality amongst bushfire fighters

Authors & year of publication	Country	Study design	Sample size	Study Participants		Exposure assessment	Health outcome and prevalence	Reported risk factors associated with morbidities
				Age in years (mean (SD))	Sex Male (%)			
(McFarlane 1989)	Australia	Retrospective cohort	• At 4 months post bushfire (n = 469) • At 11 months post bushfire (n = 395) • At 29 months post bushfire (n = 337)	38 (10.6)	NR	Bushfire fighters fought the fire for 15.6 h on average	**PTSD:** • At 4 months = 32% • At 11 months = 27% • At 29 months = 30% • One in ten bushfire fighters reported psychological morbidity in the surveys at 4,11 and 29 months post-fire	**Bushfire impact**: Only 9% of the variance in PTSD scores is due to the bushfire and other life events. More than 4-months after the bushfire, other (non-fire) factors explained the variance **Emotional distress** explains 14% of the variance in PTSD scores **Other influential factors:** individual characteristics, prior mental health conditions (Neuroticism and history of psychiatric treatment) and coping mechanisms are as influential as bushfire exposure in determining PTSD onset and progression
(Spurrell and McFarlane 1993)	Australia	Retrospective cohort study after severe bushfire event	At 42 months, selected subgroup of high-risk (130) and of 40 low-risk FF from McFarlane^35^ • n = 147 (response 84%)	NR	NR	Bushfire fighters fought the fire for 15.6 h on average	**Prevalence 42 months after bushfire fighting** • PTSD = 47.6% • Affective disorder = 29.9% • Anxiety = 46.2% Estimates not separated by high vs low risk at baseline	Risk factor: NR
(Britton et al. 2013a)	USA	Retrospective cohort	There was a total of 867 fires and 1301injuries in 5 years (2003–2007)	Age at injury = 17 to 65 years	NR	Registered wildland firefighters (data from US Department of Interior (DOI)	**Injuries** • Fractures/dislocations = 3.9% • Sprains/strains = 29.4% • Lower extremity injury = 35% • Back injuries = 10% of all injuries reported but comprised 21% of all injuries caused by equipment/tools/ machinery • Almost two-thirds of all injuries (65%) occurred during the peak season	• Mechanism of injury was associated with the type and location of the injury • Slips/trips/falls and tools were the most common mechanisms for almost half of all sprains and strains (49%) and fractures and dislocations (43%) • Injuries that occurred in the late season had higher odds of being severe compared to those in the early and peak seasons
(Britton et al. 2013b)	USA	Retrospective cohort	There was a total of 867 fires and 1301injuries in 5 years (2003–2007)	Age at injury = 17 to 65 years	NR	Registered wildland fire fighters (data from US Department of Interior (DOI))	**Injuries** Burns and heat-related injuries = 6.9% Contusions and wounds = 21.0%, Poisoning (plants)/environmental exposure = 21.6% Other unspecified injuries = 17.2%	Job tasks during fire affected risk and type of injury. 45% of engine crew injuries were sprains/strains related to slips/trips and 45% of injuries in workers performing fire suppression from the air. One-third of injuries in handcrews related to environmental poisoning /exposures. Risk of burn/smoke injuries highest in engine crews. Type 2 handcrews (hired for fire only or reassigned for the fire or on contract) were at highest odds of severe injuries
(Amster et al. 2013)	Israel	Cross-sectional	272	36.5	100%	Occupational exposure to wildfire smoke and chemical flame retardants	Burning eyes = 77% Fatigue = 71% at least 1 acute stress-related symptom after the fire = 25% (17% FF and 44% police) Persistent insomnia, bad dream and avoidance = 10% Headache = 53%	Risk factors: Police officers reported more symptoms than FF
(Leykin et al. 2013)	Israel	Cross-sectional	65	36.6 (7.9)	100%	Occupational exposure to wildfire smoke and chemical flame retardants	**One month after the fire disaster** Injuries = 6.5% PTSD = 12.3%	There was a curvilinear relationship between posttraumatic symptoms and post-traumatic growth
(Vincent et al. 2016)	Australia	Cross-shift Cohort	40	39.4(12.5)	77.5%	Multi-day bushfire fighting exposure	**Fatigue** It was higher on fire days (1.17 ± 0.17) than on non-fire days (1.24 ± 0.18) (p < 0.001)	NR
(Doley et al. 2016)	Australia	Retrospective cohort	277	42.2(10.6)	97.8%	Volunteer bushfire fighters	PTSD = 28%	Recent adverse life events and intrusive repetitive images and thoughts were significantly associated with PTSD
(Semmens et al. 2016)	Canada	Cross-sectional	499	40 (13)	83%	Occupational history as a bushfire fighter	**Different physical health conditions self-reported as diagnosed by a doctor** Basal cell skin cancer = 3%; Squamous cell skin cancer = 2%; Hearing loss = 14.4%; Asthma = 8.6%; Depression = 7.4%; Hypertension = 12.6%; Elevated cholesterol = 13.2%; Heart arrhythmia = 3.0% **Musculoskeletal surgeries (n = 499)** Back/spine = 4.8% Knee = 13.0% Ankle/foot = 5.6% Wrist/hand = 7.2%	A significant link was found between longer years as a bushfire fighter and subclinical cardiovascular conditions After adjustment for age, sex, race, household income, knee surgery risk increased with 20 + years of service (OR 3.7, 95% CI 1.1–12.5)
(Butler et al. 2017)	USA	Cross-sectional	NR	NR	NR	5 surveillance systems for fatalities (4 population-based and one case-based)	**Mortality** Between 2001 and 2012, researchers showed variance in estimated numbers of fatalities associated with WFF. When three population databases were linked, 247 unique deaths were identified amongst first responders on/around wildland fires	NR
(Glass et al. 2017)	Australia	Retrospective cohort	102,073	39.4 (14.9)	100%	Volunteer firefighters with recorded history of bushfire incidents: the number and type of incidents attended	**Mortality and cancer** • All causes of death combined = 2.71% • All types of cancer = 4.48%	**Risk factors** • Compared with the general population, there were significant decreases in overall cancer incidence and in most major cancer categories among volunteer firefighters • Prostate cancer incidence was increased compared with the general population, but this was not related to the number of incidents attended • Volunteer firefighters who attended incidents for 10–20 years had a 35% higher risk of respiratory cancer and a 36% higher risk of lung cancer compared to those with less than 10 years of service (P < 0.001) • Volunteer firefighters who attended incidents for more than 20 years had a 18% higher risk of male reproductive cancer and a 15% higher risk of prostate cancer compared to those with less than 10 years of service (P < 0.001) • The overall risk of mortality was significantly decreased, and all major causes of death were significantly reduced for volunteer firefighters There was evidence of an increased mortality from ischaemic heart disease, with increased attendance at fires
(McGillis et al. 2017)	Canada	Cross-sectional	21	29.9 (8.4)	100%	Bushfire fighters were interviewed while on duty	**Fatigue:** Self-reported fatigue was found to be prevalent among bushfire fighters	Fatigue levels were found to differ significantly between deployment types
(Stanley et al. 2018)	USA	Cross-sectional	20 (self-selected)	34.3 (10.8)	50%	Comparing bushfire fighter’s vs non-bushfire fighters	**Suicidality** Assessed with 4-item Suicidal behaviours Questionnaire-Revised (threshold of 7) Prevalence of suicidality = 55.0% in WFF and 32% in non-WFF	Lack of social connectedness may explain some of the increased risk in WFF
(Glass et al. 2019)	Australia	Retrospective cohort	16,903	38.1 (14.6)	100% Female	Firefighters with recorded history of bushfire incidents	**Mortality and cancer** • Mortality = 2.37% • Prevalence of all types of cancer = 2.4% • Breast cancer = 0.84%	**Risk Factors** Compared with the general population, female volunteer firefighters have a reduced risk of mortality. However, there was a 62% increase in the risk of female reproductive cancers for those who had served for 10–20 years compared to those who had served less than 10 years. However, for those who served for over 20 years, there was no significant difference in risk
(Moody et al. 2019)	Canada	Cross-sectional	284	> 18 years	222 males, 31 females	Forestry workers who had participated in wildfire fighting past 5 years	**Injuries** 453 injuries/illnesses reported by 56% of respondents, 88% in male respondents and most commonly (53%) in engine crew members. Sites most common: knee, back, lower leg. 22% of injuries occurred after slips/trips/falls and 90% at the fire line. 75% of injuries affected work participation that day • Joint sprain = 25.4% • Muscle strain = 15.2% • Fracture/dislocation = 7.1% • Tendinitis = 5.1% • Muscle cramp, spasm = 1.8%	**Risk factor: NR**
(Jeklin et al. 2020)	Canada	Cross-shift Cohort	30	24.6 (4.8)	66.6%	A 17-day bushfire firefighting deployment	Fatigue	Working 14 consecutive days was associated with increased levels of fatigue among bushfire fighters. Bushfire fighters reported significantly higher levels of fatigue and decreased alertness with increasing days on deployment and these levels did not improve following a three-day rest period
(Jung et al. 2021)	USA	Cross-sectional	1074	NR	Women = 100%	Firefighters Vs non-firefighters	**Miscarriage** 404 pregnancies (22%) resulted in miscarriages	Compared to a study of US nurses, firefighters had 2.33 times greater age-standardized prevalence of miscarriage (95% CI 1.96–2.75) Among bushfire fighters, volunteer firefighters had 2.53 times the risk of miscarriage (95% CI 1.35–4.78) compared to career firefighters
(García-Heras et al. 2022)	Spain	Cross-sectional	223	38 (34–45)	91.8%	Being a professional bushfire fighter	Chronic Pain (CP) = 59.7% Low back pain = 31.4%	Bushfire fighters who reported CP were significantly older (p = 0.001) and experienced (p = 0.001) than those without CP.)
(Jung et al. 2023)	USA	Cross-sectional	1074	40.0 (8.2)	Women = 100%	Firefighters Vs non-firefighters	Preterm Birth = 12%	Among firefighters, the prevalence of preterm birth was 1.41 times higher than non-firefighters Volunteer firefighters face a greater risk of preterm birth compared to career firefighters
(Psarros et al. 2018)	Greek	Cross-sectional	102	35.1(10.6)	100%	Being on duty during the entire period of the bushfire fighting	PTSD = 18.6%	Individuals with PTSD were more likely to be younger, seasonal firefighters, and have higher anxiety and less experience dealing with disasters than those who did not
(Becker et al. 2023)	Portugal	Cross-sectional	116	34.8 (10.4)	75%	Self-selected firefighters who battled violent forest fires	PTSD using PCL-5 measure (threshold not stated) = 12.7% Somatic Symptom Disorder = 3.9% and “psychopathologies” defined from Depression, Anxiety and Stress Scale (DASS-21) and Brief Symptom Inventory (BSI)	Female gender was associated with SSD (r = 0.344, p < 0.001) and psychopathologies (r = 0.290, p < 0.001) Both SSD and psychopathologies mediate the relationship between Post-traumatic experience and PTSD
(Hasan et al. 2023a)	USA	Cross-sectional	11,051	17–29(38.10%) 30–39 (32.80%) 40–49 (18.50%) 50–65 (10.50%	85.3%	Federal WFFs employed	**Hypertension** Bush firefighters = 6.2% Weighted USA Population = 5.8%	WFFs had more than double likelihood/odds of hypertension than the US general population
(Hasan et al. 2023b)	USA	Cross-sectional	2862	17–29 (48.2%) 30–39 (37.0%) 40–49 (9.8%) 50–65 (4.1%)	86.3%	WFF with a prior work history assigned on fire for arduous study	32% of WFFs had one or more clinical measures that would place them in high-risk categories for body mass index, blood pressure, and total cholesterol	Obesity(OR = 1.4, 95%CI = 1.2, 1.8), hypercholesterolemia (OR = 1.2,95%CI = 1.0, 1.5)—associated with WFF days assigned on fire, (highest versus lowest tertile of days on fire) Hypertension, prehypertension- not associated with days assigned on fire for WFFs
(Berecki-Gisolf et al. 2024)	Australia	Cross-sectional	44,164 claims	< 25 (4.1%) 25–34 (25.0%) 35–44 (29.9%) 45–54 (28.7%) ≥ 55 (12.2%)	74.9%	Workers compensation claims of ambulance officers, paramedics, police, firefighters. Extreme bushfire claims—claims with injury/disease date coincided with Black Saturday, Black Summer bushfires in Victoria	2.5% had recorded injury/disease onset dates within extreme bushfire periods	Extreme bushfire period claims were associated with older workers (OR = 1.58,95%CI = 1.30–1.92, ages ≥ 55 vs. 35–44 years). Mental disorders (OR = 1.61,95%CI = 1.25–2.07), intracranial injuries (OR = 3.04,95%CI = 1.69–5.48) and infections/parasites (OR = 3.11,95%CI = 1.61–5.98) vs. wounds were associated with extreme bushfire period claims
(Wah et al. 2024)	Australia	Cross-sectional	120,022 claims	< 25 (8.5%) 25–34 (21.5%) 35–44 (24.8%) 45–54 (27.1%) ≥ 55 (18.1%)	68.3%	Workers compensation claims of ambulance officers, paramedics, police, firefighters, other occupations Extreme bushfire claims—claims with injury/disease date coincided with Black Saturday, Black Summer bushfires in Victoria	Crude, age-standardised rates of all injury/disease, including mental, musculoskeletal, respiratory claims -elevated among 3 emergency responders groups and other occupations during extreme bushfire periods compared to off-seasons, summer	All first responders- higher rates of mental health claims in extreme bushfires compared to off-seasons relative to other occupations Firefighters—higher rates of mental health claims in extreme bushfires compared to off-seasons (IRR = 3.9, 95%CI = 2.28–6.67), summers (IRR = 2.43, 95% CI = 1.39–4.25), relative to other occupations
(Jaiswal et al. 2024)	Australia	Cross-sectional	338	18–29(17%) 30–39(22%) 40–49(28%) 50–59(24%) 60–69(8%) 70 + (1%)	78%	Employed/volunteer EMS personnel with history of smoke exposure through emergency wildfire /planned burn	Eye irritation -90% of firefighters at least sometimes during work, 70% after work	Frequency of eye irritation-greater amongst females than males (OR 2.01, CI 1.22–3.31)
(García-Heras et al. 2024)	Spain	Cross-sectional	217	36.3 (6.3)	91%	Brigadas de Refuerzo contra Incendios ForestalesWFF	Injuries -76% amongst 271 respondents (eligible 544 in total)	higher propensity for injury among those aged ≥ 35 (OR = 2.14, CI = 1.12–4.06, with > 10 years’ experience (OR = 2.46, CI = 1.30–4.67) Injuries occurred mainly during physical training (46%), preventive work(33%) forest fires (20%)

*SD* Standard deviation, *NR* Not reported, *PTSD* Post-traumatic stress disorder, *MSK* Musculoskeletal injuries, *FF* Firefighters, *WFF* Wildland firefighters, *CP* Chronic pain, *PCL-5* PTSD Checklist-5, *SSD* Somatic symptom disorder, *DASS-21* Depression, Anxiety and Stress Scale, *BSI* Brief symptom inventory, *OR* Odds ratio, *95% CI* 95% confidence interval, *IRR* Incidence rate ratio, *EMS* Emergency medical service

### Mental health conditions and suicide

In total, eleven studies evaluated mental health (Stanley et al. 2018; McFarlane 1989). (Spurrell and McFarlane 1993; Doley et al. 2016; Semmens et al. 2016; Amster et al. 2013; Leykin et al. 2013; Becker et al. 2023; Psarros et al. 2018; Berecki-Gisolf et al. 2024; Wah et al. 2024), none of which were rated high quality and four of which were rated moderate quality; (McFarlane 1989; Becker et al. 2023; Berecki-Gisolf et al. 2024; Wah et al. 2024). Different measures were used: three studied assessed self-reported symptoms using the 12-item General Health Questionnaire (GHQ). (McFarlane 1989; Spurrell and McFarlane 1993; Doley et al. 2016) Six studies explored the risk of post-traumatic stress disorder (PTSD) using self-reported screening questionnaires (McFarlane 1989; Spurrell and McFarlane 1993; Doley et al. 2016; Leykin et al. 2013; Becker et al. 2023; Psarros et al. 2018). Two studies evaluated workers’ compensation claims (Berecki-Gisolf et al. 2024; Wah et al. 2024). One study explored rates of suicide (Stanley et al. 2018).

In 1989, McFarlane et al. followed up 469 firefighters after a devastating Australian bushfire which caused injury to 27%, hospital admission for 12%, bereavement of 7% and personal property damage to 23%. All were screened for PTSD using the GHQ, and 8 months after the fire, 50 firefighters underwent an interview using the diagnostic criteria listed in DSM-III(Diagnostic and Statistical Manual of Mental Disorders) at the time for PTSD to confirm the diagnostic utility of the screening tool. Retention was 84% and 72% at 11 and 29 months (Jung et al. 2021). Based on a 50% cut-off on the GHQ scores, the estimated prevalence of PTSD was 32% at 4 months; 27% at 11 months; and 30% at 29 months (McFarlane 1989). Subsequently, 147 of the original firefighters were followed up (Spurrell and McFarlane 1993). All individuals at high risk of PTSD from prior screening at 4-, 8- and 29 months and a smaller sample of low-risk individuals were interviewed. Their results were not separated by prior risk status, however. Consequently, the prevalence rates reported: PTSD 47.6%, affective disorder 29.9%, and anxiety 46.2%, with considerable overlap of the three diagnoses, represent the “worst case” (Spurrell and McFarlane 1993). After a severe Israeli forest fire in 2010, 204 firefighters and 68 police officers volunteered for a study a few months later and 25% reported that they had experienced acute stress-related symptoms after the fire (Amster et al. 2013).

Also after a forest fire, Becker et al. reported that 12.7% of 116 Portuguese volunteer firefighters involved in firefighting and volunteering to complete questionnaires had scores on the PTSD Checklist-5 (PCL-5) measure consistent with PTSD (although the cut-off chosen was not specified) (Becker et al. 2023).

Three low-quality studies also reported the prevalence of mental health conditions (Spurrell and McFarlane 1993; Semmens et al. 2016; Stanley et al. 2018). As part of a wider health survey amongst wildfire fighters in USA, 7.4% of 499 survey respondents reported that they had been diagnosed with depression by a doctor with the highest rates (9.6%) amongst those who had been wildland firefighting for 10–19 years (3.3% amongst those fighting < 10 years and 7% amongst those fighting 20 + years) (Semmens et al. 2016). One study used a merged dataset from two national studies of firefighter mental health (n = 1131) amongst whom 1.8% (n = 20) were wildland firefighters. Based on responses to the Suicidal Behaviours Questionnaire-Revised 4-item questionnaire, their suicidality was assessed using a cut-off threshold of 7. Comparing scores for wildland as compared with non-wildland firefighters, a larger proportion of wildland firefighters (55% vs 32%) were self-reporting suicidality after controlling for sex (Stanley et al. 2018). However, given the very small sample size, and self-selection for participation, these findings should be interpreted with caution.

Two Australian studies evaluated workers’ compensation data and found that first responder claims for mental health disorders increased during extreme bushfire periods relative to summer and off-season (Berecki-Gisolf et al. 2024; Wah et al. 2024). Specifically in firefighters, claims for mental health increased almost fourfold relative to off-season (IRR 3.9, 95% CI 2.3–6.7) (Wah et al. 2024).

The risk factors associated with PTSD among bushfire fighters and other emergency responders have been studied, including personal, work-related, and bushfire factors. McFarlane et al. found that bushfire exposure explained 9% of the variance in PTSD scores 4-months after the fire, but other factors explained the variance seen after 11-, 29- and 42-months. Emotional distress during events accounted for an additional 14% (McFarlane 1987). In other studies, personal characteristics, prior mental health conditions, and coping mechanisms were evaluated but using heterogeneous methodology (Stanley et al. 2018; McFarlane 1989, 1987, 1988b, 1988a, 1988c; Doley et al. 2016; Psarros et al. 2018).

### Injuries

Britton et al. studied the epidemiology of injuries relating to bushfire fighting 2003–2007 in the USA, reporting 1301 non-fatal injuries during this period (Britton et al. 2013a, 2013b). The most common mechanism of injury was slips/trips and falls (28%), followed by injuries from equipment /tools /machinery (22%) (Britton et al. 2013a). Most injuries occurred during peak fire season (65%). Over half (55%) of fire/smoke/flash burn injuries occurred in the early season. As expected, slips/trips/falls were associated with half of all sprains/strains (49%) and 43% of fractures/dislocations. The most commonly injured musculoskeletal site was the lower limb, while back injuries accounted for 10% of the total injuries, although they represented one in five injuries caused by equipment, tools, or machinery(Britton et al. 2013a). In total, 14% of the non-fatal injuries were “severe” (defined as necessitating lost work days, restricted activities at work or job transfer) and the factors associated with the highest rate of severe injuries were equipment/tools /machinery and “weather-related”. Exploring by job allocation during the fires, engine crews (workers who provide water, hoses and pumping capability to a fire) experienced one-third of all injuries (33.6%) (45% of which were sprains and strains), and both cause and nature of injuries sustained were related to job tasks. One-third of injuries amongst hand crews (workers using shovels, rakes and saws to clear vegetation around the fire) were poisoning (from plants) and/or related to other environmental exposures. Engine crews were at the highest risk of injuries from burns and smoke (Britton et al. 2013b).

Also in the USA, Moody et al. undertook an electronic survey of injury amongst US Forest Service permanent or seasonal employees who had worked in at least one wildfire in the preceding 5 years, using snowball sampling. Amongst 284 respondents (estimated response rate 35%), 157 respondents (56%) reported multiple injury/illness and total 453 injury/illnesses were reported over 5 years. 254 respondents reported at least 1 injury/illness, 88% of which occurred in male respondents and 53% of all injuries were reported by engine crew members. Types of injury in order of most frequent were: joint sprains (25.4%), muscle strains (15.2%), fractures or dislocations (7.1%), tendonitis (5.1%), and muscle cramps or spasms (1.8%) (Moody et al. 2019). The most common site of injury was the knee (17%), back (16%), and lower leg (13%). Slips/trips/falls accounted for 22% of injuries but “over-exertion” accounted for 12.5%. Over 90% of injuries occurred at the fire line and over 75% impacted the worker’s ability to firefight that day. Unfortunately, given that the study aims were clearly stated, participation bias is highly likely in this study (Moody et al. 2019).

In another cross-sectional survey, this time in Spain, 38% of a group of specialist wildland firefighters completed a questionnaire about occupational injuries. Amongst respondents, most (76%) had sustained an injury with a higher risk amongst older bushfire fighters (aged > 35 years) with more than 10 years’ of experience. A high proportion of injuries occurred whilst physical training (over half of all injuries amongst leaders) and most were musculoskeletal with lower limbs most commonly involved (García-Heras et al. 2024).

Analysis of workers’ compensation claims data from Australia also showed a spike in first responder claims for musculoskeletal claims during extreme bushfire periods as compared with summer or off-season. Firefighters had 1.5 times higher claims rates for musculoskeletal injuries in summer and extreme bushfires than off-seasons compared with other occupational groups (Wah et al. 2024).

In another analysis of workers’ compensation data, Berecki-Gisolf et al. showed that, during extreme bushfire periods, claims for intracranial injuries were increased amongst first responders relative to off-seasons (OR 3.04, 95%CI 1.7–5.5) (Berecki-Gisolf et al. 2024).

### Arthritis and Musculoskeletal surgery

In their cross-sectional survey, Semmens et al. asked about doctor-diagnosed arthritis (excluding rheumatoid arthritis) and operations to the back/spine, wrist/hand, knee, and ankle/foot (Semmens et al. 2016). Three per cent of wildfire fighters who had served < 10 years, 9% who had served 10–19 years and 17% who had served 20 + years reported doctor-diagnosed arthritis, but this was not a significant trend by the duration of service. In total, 24 reported back/spine operations, 65 knee operations, 28 ankle/foot operations and 36 wrist/hand operations (Semmens et al. 2016). After adjustment for age, sex, race and household income, knee surgery was more frequent in firefighters with 20 + years of service (OR 3.7, 95%CI 1.1–12.5), albeit with wide confidence intervals, given the small numbers.

### Chronic pain

In a study of chronic pain amongst bushfire fighters, García-Heras and colleagues sent a 45-item questionnaire to all members of Spanish Forest Fire Reinforcement Brigades, elite bushfire fighters who are urgently conveyed to bushfire sites during events (García-Heras et al. 2022). There were 221 respondents (including 18 females, which was 100% of eligible sample) (38% response amongst males). In total, 59.7% reported chronic pain (persistent pain > 3 months), with increased prevalence in older respondents. Just under half (45.5%) of those with chronic pain had single-site pain. Increasing age and increasing years of experience were associated with the risk of chronic pain (García-Heras et al. 2022). Widespread chronic pain (upper and lower limbs and spine) was the most common distribution. Participation bias was also likely in this study.

### Cancer

Three papers investigated cancer risk among bushfire fighters (Glass et al. 2017, 2019; Semmens et al. 2016). Glass et al. linked national firefighter data for Australian male volunteer firefighters (aged on average 48.7 years) to explore cancer risk. In total, the cohort had 7057 cancers diagnosed amongst 163,094 male volunteer firefighters. Both the overall incidence of cancer (Standardized Incidence Rate [SIR]: 0.59; 95% CI: 0.55–0.62) and the incidence rates for the major different types of cancer were significantly lower in the volunteer firefighters (Glass et al. 2017). The overall risk of cancer was not found to increase by increasing numbers of fire events attended, but the incidence of kidney cancer increased with attendance at landscape fires (Glass et al. 2017). Overall risk of lung cancer was significantly reduced among volunteers and length of service was associated with a greater reduction in risk of lung cancer. However, rates of prostate cancer were found in excess among volunteer firefighters, with risks which increased with years of service but not the number of events attended. An excess of prostate cancer has also been reported in career firefighters, but it is unclear if the excess can be explained by attendance for screening /establishing a diagnosis amongst firefighters or a real increased risk. As Glass and colleagues pointed out, however, the cohort of volunteer firefighters was young at the time of the linkage (mean age under 50 years), and most cohort members had served for less than 10 years, so a later risk of some types of cancer cannot be ruled out.

In another study by Glass et al., the risk of cancer was studied in 37,973 Australian female volunteer firefighters (mean age 46 years), 44% of whom were recorded as having ever attended fire incidents. There were 1027 cancers in total, with risk of all types of cancer similar to that of the background population (SIR: 0.97; 95% CI 0.88–1.07) (Glass et al. 2019). Rates of lung cancer were (non-significantly) increased among those females who had attended fires (SIR 1.3, 95%CI 0.9–1.82). Breast cancer risk was not found to be increased. Comparing females with the longest duration of 10–20 years of service with those with < 10 years of service, there was an increased risk of female reproductive cancers with increasing fire attendance.

In their survey, Semmens et al. also enquired about cancer among the 499 wildfire fighters; mean age was 40 years (Semmens et al. 2016). Responses suggested that the prevalence of all cancer types was below 1%, except for skin cancers: basal cell cancer (3%) and squamous cell cancer (2%) (Semmens et al. 2016). With such small numbers and a very young cohort, no further analyses could be performed.

### Cardiovascular diseases, arrhythmias, hypertension and hypercholesterolaemia (CVD)

Three studies considered cardiovascular health (Hasan et al. 2023a, 2023b; Semmens et al. 2016). Hasan et al. explored subclinical measures of cardiovascular health, including body mass index (BMI), total cholesterol, smoking, and blood pressure amongst 11,051 wild firefighters aged 17–65 years. Data collected on routine examinations among firefighters were compared with data from the National Health and Nutrition Examination Survey (NHANES). Unfortunately, the NHANES dataset differed in terms of race, gender and age distribution (firefighters younger, more likely male, less racial variation). Whilst BMI, the proportion of current smokers, diagnosis of diabetes and total cholesterol were higher in the NHANES sample, the proportion of firefighters with hypertension was higher (6.2% vs 5.8%) among firefighters (Hasan et al. 2023a). Unfortunately, given the variance with the comparison group selected and the fact that firefighters' blood pressure was recorded routinely once per year and NHANES' blood pressure was measured three times in clinical conditions, it is difficult to know how important this finding is. Another study by Hasan et al. (2023b), a cross-sectional analysis of 2,862 wild firefighters with a history of arduous fire assignments, found that greater exposure to fire duty (highest versus lowest tertile of days assigned on fire) was significantly associated with 1.4- and 1.2-times increased odds of obesity and hypercholesterolemia (Hasan et al. 2023b). However, no significant association was observed between fire duty days and hypertension or prehypertension.

In a cross-sectional survey of self-reported symptoms, 13% of the 499 responding wildland firefighters reported doctor-diagnosed hypertension, 13% raised cholesterol, and 3% heart arrhythmia (Semmens et al. 2016). High serum cholesterol was reported by 3% of bushfire fighters with less than 10 years of experience, 10% among those with 10 to 19 years, and 29% among those with more than 19 years of experience. Arrhythmias were not reported by bushfire fighters with less than 10 years of experience, 2% of those with 10 to 19 years, and 7% among those with more than 19 years of experience. Of these, after adjustment for age, sex, and race, there was a trend for the risk of hypertension to increase with years of service but with wide confidence intervals due to the small numbers (Semmens et al. 2016).

### Reproductive outcomes

Two studies explored birth outcomes among female bushfire fighters (Jung et al. 2021, 2023), Jung et al. (2021) aimed to compare the risk of miscarriage amongst female firefighters doing different types of firefighting (career/volunteer; wildland vs structural) and compare the risk of miscarriage amongst female firefighters with that seen in female nurses (Jung et al. 2021). Female firefighters (n = 3181) were recruited to a general health study by snowball sampling methods. The researchers reported that, among 1074 female firefighters and 1864 total pregnancies, 404 (22%) pregnancies resulted in miscarriage, which compared with nurses participating in the Nurses’ Health Study II, suggested a 2.33 times greater age-standardized risk of miscarriage (95% CI 1.96–2.75). Unfortunately, the authors did not present a comparison of the characteristics of responding firefighters as compared with participants in the Nurses’ Study, but they are likely to differ somewhat, and it is impossible to rule out participation bias amongst the firefighters. Comparing responding firefighters, the authors found that 45% of volunteer firefighters reported a history of miscarriage as compared with 28% of career firefighters. Exploring by exposure to wildland or wildland-urban interface fires (n = 363), as compared with structural firefighting (n = 709), rates of reported miscarriage were 39% vs 14% respectively and volunteer firefighters had a 2.5-fold increased risk. Given the study's limitations, these findings need to be interpreted cautiously.

In their second study, Jung et al. compared the reported prevalence of pre-term births amongst female firefighters in the same study sample as that described above, comparing with US general population data and the Nurses’ Health Study (Jung et al. 2023). Rates of pre-term birth were reported to be increased among participating female firefighters when compared with US women in the general population and in the Nurses’ Health Study. Here again, however, participation bias cannot be excluded. An increased risk of preterm birth was also reported amongst volunteers as compared with career firefighters (Jung et al. 2023). However, no differences were seen amongst female firefighters exposed to wildland or wildland-urban interface firefighting, as compared with US women.

Further exploration of the self-reported data suggested that firefighters who started restricting their work in the 2nd trimester had a somewhat reduced risk of preterm birth (Relative Risk: 0.67, 95% CI: 0.43–1.03) than those who started reducing work in the 3rd trimester or did not restrict work at all (Jung et al. 2023). Given the small sample sizes, self-reported data and risk of participation bias, these results need to be interpreted cautiously.

### Sleep and Fatigue

Four studies have evaluated sleep and fatigue in bushfire fighters (Vincent et al. 2016; McGillis et al. 2017; Jeklin et al. 2020; Amster et al. 2013). The first, among 40 Australian firefighters over 4 weeks during a multi-day bushfire, evaluated work diaries and data from wrist-worn work activity monitors over at least two shifts and up to 9 shifts in total (a total of 205 shifts recorded). Time in bed and total sleep time was reduced by, on average, an hour during fire days but with a large inter-individual variation. When sleeping in a tent or vehicle, sleep time was more reduced than when sleeping at home/motel. Sleep time was also reduced after longer shifts (> 14 h). None of the sleep efficiency, latency, or subjective sleep quality varied by fire days vs non-fire days. Pre- and post-sleep fatigue were increased on fire days. In a similar study in Canada, 21 male bush firefighters were recruited, amongst whom 11 complied with diaries and wrist monitors consistently (McGillis et al. 2017). Sleep duration and sleep quality were generally poorer than that recommended and were most restricted during Initial Attack fires (defined as the action taken to halt the spread arriving first at the fire), which were also associated with more fatigue. However, total sleep time was not affected by the duration of work periods or shift length, nor was there an impact on shift times. Similar measures were assessed during a period of 14-day bushfire deployment and 3 post-deployment days amongst 30 firefighters (Jeklin et al. 2020). Measures of sleep and fatigue were assessed immediately after returning to base on fire days, at the same time as wrist-based activity monitors were put on, supervised by researchers. Data from 416 shifts were analysed and again demonstrated that total sleep time was suboptimal throughout in all participants. Objective cognitive performance did not change, but firefighters had slower reaction times on day 14 than on day 1. They also had more subjective fatigue on day 13 than on day 3 and day 16 compared to day 5. Total sleep time was reduced by an average of 10 min over the fire days and was around 10 min longer on rest days than fire days. Sleep efficiency and latency did not change but sleep quality was assessed as better during deployment than on rest days. Somnolence was greater on day 16 than on day 1. In a fourth study, during the severe bushfire of 2010 in Israel, 272 first responders (204 firefighters and 68 police officers) reported 18.4 h and 11.9 h of working without sleep and, consequently, increased symptoms of self-assessed fatigue during the fire, as compared with after, the fire.(Amster et al. 2013).

### Other health impacts

In the cross-sectional USA study, 14.4% (72/499) of wildland firefighters self-reported doctor-diagnosed hearing loss (Semmens et al. 2016), which was not associated with a longer duration of service. During and after the forest fire, firefighters reported an increased frequency of burning eyes (Amster et al. 2013) and eye irritation (Jaiswal et al. 2024). Firefighters reported that protective eyewear helped to decrease eye symptoms (Jaiswal et al. 2024). However, its consistent use on the ground was challenging and could affect operational capabilities.

### Mortality

Acute mortality related to bushfire fighting was measured in the USA from 2001–2012, deriving data from 5 different surveillance databases (4 population databases and one case-based system) to identify deaths. The authors pointed out that the derived data gave different estimated mortality rates (by 10% or so). Three of the population-based systems were then linked and in total, 247 deaths were identified, 73% of which were recorded in all three databases (Butler et al. 2017). Most (94%) deaths occurred amongst males, more than 55% of whom were aged > 40 years, and one-third were volunteer firefighters (the single largest group). The most common causes of death were incidents associated with aviation, vehicles, medical events, and entrapments/turnovers (Butler et al. 2017). However, the study could not take account of the total number of individuals involved in wildland-fire-related activities, making it impossible to calculate mortality rates. Additionally, this surveillance approach could only account for fatalities during or on the way to/from a fire.

In an Australian linkage study including data from 102,073 male volunteer firefighters who had ever attended fire incidents, Glass et al. reported that 2,768 deaths were observed, resulting in a Standardised Mortality Ratio (SMR) of 0.52 (95% CI: 0.50 to 0.53), which indicates a 48% lower mortality rate compared to the general population (Glass et al. 2017). Except for fire-related deaths, volunteer firefighters had lower mortality from all common causes, including lung cancer. Internal analyses showed increased relative mortality rates (RMRs) for ischaemic heart disease amongst those who attended most commonly at incidents and attendance at bushfires (RMR 1.54, 95% CI 1.06–2.23). The decreased SMRs were attributed to the healthy worker effect, i.e. selection to become a firefighter.

In another linkage study including data about 16,903 Australian female volunteer firefighters who had ever attended fire incidents, Glass et al. observed 206 deaths (SMR 0.72 (95% CI: 0.62- 0.82) (Glass et al. 2019). Apart from accidental deaths, female volunteer firefighters had lower SMRs for all causes of death. Considering RMRs, women who attended forest fires most often had a higher RMR for all malignancies (RMR 1.52, 95%CI 1.07–2.15) but not for other causes.

## Discussion

This systematic review identified 27 papers which examined the non-respiratory health impacts and risk of mortality associated with bushfire fighting. The overall quality of the included studies was low, with sixteen studies rated as poor and eleven as moderate quality, with most rated down because of inadequate control for potential confounders and a reliance on self-reported data for both exposure and health outcomes. The strongest body of evidence was seen for an increased risk of mental health conditions, including PTSD. Injuries were also reported relatively commonly, and there were more than 247 acute bushfire-related deaths in the USA over 12 years. There was convincing evidence from two large-scale linkage studies that volunteer firefighters, at least when in their late forties, involved in bushfires have lower all-cause mortality and cancer risk than the general population. Whilst the review aimed to explore longer-term health impacts, there was far less long-term data than there was data about shorter-term health impacts, such as mental health conditions and injuries.

There is growing evidence of an association between mental health conditions and bushfire fighting, including PTSD, stress, anxiety, and depression, although, for the most part, causal inferences cannot be drawn from the current evidence, and longer-term follow-up studies are lacking. In many cases, PTSD symptoms are self-reported according to symptom questionnaires, which are thought to lead to higher prevalence estimates than objective assessments (Levis et al. 2020). Longer-term follow-up studies are required to determine natural history and risk of progression, but importantly, the data need to be interpreted alongside information about attendance at subsequent bushfires during the follow-up period (McFarlane 1989, 1986). Some studies evaluated risk factors for PTSD, including individual characteristics, introversion, neuroticism, prior mental health conditions, emotional distress, and coping mechanisms, (McFarlane 1989, 1988b, 1988a, 1988c) but the lack of high-quality prospective studies with good data about exposures (e.g. hours worked, traumatic events witnessed) and confounding factors (history of mental health conditions, prior experience of trauma) is hampering the development of preventive strategies. Mental health conditions result in major impacts on the individual and their family and may keep them away from their paid occupation for longer than physical health conditions(Collie et al. 2016) therefore better research is needed to understand the relationship with repeat bushfires and how to best prevent and/or provide long-term support and interventions to address the mental health needs of bushfire fighters.

Unsurprisingly, bushfire fighting was associated with an increased risk of injuries, including burns, contusions, poisonings, sprains, strains, and fractures. Among these, musculoskeletal injuries were common (Orr et al., 2019), specifically sprains and strains, mostly caused by slips, trips, and falls. In an elite group of Spanish bushfire fighters, injuries were most common during physical training and preventive work (García-Heras et al. 2024). However, the wide range of injuries underscores the significance of customised preventive measures and targeted interventions to lower the risk of injury. Recent workers’ compensation data highlighted an increased risk of injuries during extreme bushfire events amongst older first responders, possibly related to reduced fitness, poorer coordination or lack of training.(Berecki-Gisolf et al. 2024) Specifically, efforts should focus on evaluating and, where necessary, enhancing existing safety protocols, comprehensive training programs, and the proper use of personal protective equipment. Although the studies were limited, there was some evidence of increased CVD mortality among male bushfire fighters and other emergency responders, together with a possible excess of cardiovascular risk factors (Hasan et al. 2023a, 2023b; Semmens et al. 2016). This is potentially important, given an increased risk of cardiovascular risk factors amongst career firefighters (Smith et al. 2020; Bode et al. 2021) and the excessively high rate of mortality from on-duty sudden cardiac deaths amongst career firefighters (Smith et al. 2019). Firefighting can lead to significant cardiovascular strain, including alterations in cardiac function, vascular function, and haemostasis (Smith et al. 2016). The strain can result from sympathetic nervous system activation, strenuous physical work, exposure to Polypropylene Carbonate, and/or carbon monoxide and smoke exposure. Particulate matter in smoke (Dennekamp et al. 2015) or diesel engine exhaust (Mills et al. 2007; Cosselman et al. 2012) are known to be associated with an increased risk of heart attacks. If the same risk is observable in bushfire fighters, it is important to identify strategies to mitigate these risks for these vital workers.

Other studies in this review point to the important adverse effects of bushfire fighting on sleep and the risk of fatigue. Fire days were associated with excess fatigue compared to non-fire days and fatigue increased with days spent firefighting (Jeklin et al. 2020). The demanding and stressful nature of firefighting, especially during active fires, often leads to disrupted sleep patterns and inadequate rest (Cuenca-Lozano and Ramírez-García, 2023; Aisbett and Nichols 2007). Incorporating assessments of sleep quality and quantity, including factors such as irregular/long working hours and sleeping conditions in camps or while on the move, into future research on bushfire fighters could provide valuable insights into strategies for reducing fatigue and its associated risks.

It has been recognised in population studies that pregnancy exposure to pollutants, including PM_2.5_ is associated with an increased risk of adverse pregnancy outcomes, including pre-term birth, low and high birthweight, gestational diabetes and congenital anomalies (Amjad et al. 2021) Only two studies evaluated birth outcomes in female bush firefighters (Jung et al. 2021, 2023) and suggested increased risks of miscarriages (Jung et al. 2021) and preterm births (Jung et al. 2023). Both studies were, however, small and cross-sectional, with methodological challenges, such as failure to account for confounding factors like socioeconomic status, lifestyle factors (e.g., smoking and alcohol consumption), pre-existing health conditions, and access to prenatal care (Sadiq et al. 2016). Also, in both cases, the choice of the referent population may not have been ideal. Future studies should aim to include larger sample sizes and a thorough consideration of potential confounding factors to better understand the impact of bushfire exposure (particularly cumulative exposure) on the health of the women and their babies.

The overall quality level of included studies was low (sum of all scores 195/27 = 7 = low), indicating limitations in design and methodology. Most studies followed a retrospective design or were cross-sectional, neither of which facilitated answering important research questions due to their increased risk of bias and inability to clarify causality (Thiese 2014). Sample sizes were also generally quite small, given the methodological challenges of surveying this relatively itinerant workforce who are frequently volunteers from other occupational backgrounds. There is a need for collecting prospective, high-quality data over longer time periods to address some of the critical research questions.

### Strengths and limitations of the review

One of the major strengths of this review is the use of a comprehensive search strategy, which yielded a large number of records. The screening process was conducted independently by two authors, enhancing the reliability of the study selection.

However, several limitations should be noted. First, the review included only articles published in English, raising the possibility that relevant studies published in other languages were missed. Second, the included studies exhibited significant heterogeneity in exposure definitions, intensity, and duration of bushfire fighting, as well as differences in outcome measurement tools and statistical methods. There was a high dependence on self-reported exposures and outcomes. Additionally, many of the included studies lacked an appropriate control or non-exposed group or used another worker population which was not necessarily comparable (e.g. nurses in the Nurses Health Study or participants in NHANES). Two studies adopted a cross-shift cohort design, comparing health outcomes before and after bushfire fighting periods (Vincent et al. 2016; Jeklin et al. 2020). While this approach captures short-term outcomes such as injuries, it is inadequate for examining long-term conditions, including CVD, cancer, and PTSD. Thus, future research should prioritise high-quality, longitudinal studies with appropriate comparison groups to better understand the long-term health risks associated with bushfire fighting.

## Conclusion

This review identified 27 studies of health and mortality related to bushfire fighting. Evidence is growing for an association with mental health conditions and there is unsurprisingly an increased risk of injuries. However, more methodologically rigorous research is needed to clarify the links between occupational bushfire exposure and other long-term health risks. Future studies should prioritise longitudinal designs and include suitable comparison groups whilst more carefully controlling other confounding influences, including lifetime occupational history and individual exposure to pollutants.

## Supplementary Information

Below is the link to the electronic supplementary material.

Supplementary file1 (DOCX 28 KB)

## Data Availability

This study is a systematic review of published literature. All data analysed during this review are included in the published articles cited in the manuscript. No new datasets were generated or analysed by the authors. Supplementary materials, including the full search strategy and quality assessment details, are available in Appendix A.
