# Supplementary material for: Non-respiratory health risks and mortality associated with fighting bushfires (wildfires): a systematic review
Source: Int Arch Occup Environ Health. 2025 Apr 9;98(4-5):343–67. doi: 10.1007/s00420-025-02138-7 (PMC12238121; doi:10.1007/s00420-025-02138-7)
Supplement: Supplementary file 1 — Supplementary file1 (DOCX 28 KB) [file 420_2025_2138_MOESM1_ESM.docx]

**Appendix 1 Search strategies**

| **No** | **Medline** |
| --- | --- |
| 1 | exp Fires/ or bushfire.mp. |
| 2 | Wildfires/ |
| 3 | Fires/ or exp Wildfires/ or Wildfire*.mp. |
| 4 | (bushfire* or bush fire* or wildfire* or wild* fire* or forest fire* or wildland fire* or wild land fire* or woodland fire* or wood land fire* or brushfire* or brush fire* or rural fire* or grassfire* or grass fire or vegetation fire* or landscape fire*).mp. [mp=title, book title, abstract, original title, name of substance word, subject heading word, floating sub-heading word, keyword heading word, organism supplementary concept word, protocol supplementary concept word, rare disease supplementary concept word, unique identifier, synonyms, population supplementary concept word, anatomy supplementary concept word] |
| 5 | Fires/ and (bush* or biomass or forest* or grass* or habitat* or vegetation or wild* or ecosystem* or savanna* or agricultur* or prescribed burn* or prescribed fire*).ti,ab,kw,kf. |
| 6 | (bushfire* or forestfire* or grassfire* or wildfire* or smoke pollution event* or (fire* adj3 (bush* or biomass or forest* or grass* or habitat* or vegetation or wild* or ecosystem* or savanna* or agricultur*)) or prescribed burn* or prescribed fire*).ti,ab,kw,kf. |
| 7 | 1 or 2 or 3 or 4 or 5 or 6 |
| 8 | morbidity/ or mortality/ or "cause of death"/ |
| 9 | patient care/ or hospitalization/ or patient admission/ or patient discharge/ or patient readmission/ |
| 10 | patients/ or inpatients/ |
| 11 | exp disease/ or exp disease attributes/ |
| 12 | emergency service, hospital/ or trauma centers/ |
| 13 | exp Emergency Medical Services/ |
| 14 | Ambulances/ or Air Ambulances/ |
| 15 | exp "Wounds and Injuries"/ |
| 16 | exp "diseases (non mesh)"/ or exp respiratory tract diseases/ or bronchial diseases/ or exp asthma/ or exp lung diseases/ or exp respiration disorders/ |
| 17 | exp Sleep Wake Disorders/ |
| 18 | mental health/ or mental disorders/ or exp anxiety disorders/ or exp mood disorders/ or exp "trauma and stressor related disorders"/ |
| 19 | mental health services/ or psychological problem/ or psychiatric problem*/ or occupational stress*/ or psychological distress/ or post-traumatic stress disorder/ or PTSD/ or mental fatigue/ or Burnout/ or Emotional exhaustion/ or Sleep disturbance*/ or Substance abuse/ or Job-related stressor*/ or Traumatic exposure*/ or Cumulative stress/ |
| 20 | exp pregnancy/ or exp pregnancy outcome/ |
| 21 | exp pregnancy complications/ or exp cardiovascular diseases/ |
| 22 | (health* or disease* or mortalit* or morbidit* or incidence* or hospital* or admission* or injur* or traum* or emergency or emergencies or ambulanc*).mp. |
| 23 | (asthm* or cardio* or allerg* or respirator* or COPD or lung diseas* or lung function* or mental health* or sleep* or disorder* or insomni*).mp. |
| 24 | (pregnan* or gestation* or maternal or preterm* or pre term* or pre matur* or prematur* or post matur* or postmatur* or abortion* or stillbirth* or still birth*).mp. |
| 25 | neoplasms/ or neoplasms, post-traumatic/ or eye diseases/ or cardiovascular diseases/ or immune system diseases/ or occupational diseases/ or "wounds and injuries"/ |
| 26 | (physiological adj3 response*).mp. |
| 27 | (Physiological adj3 well-being).mp. [mp=title, book title, abstract, original title, name of substance word, subject heading word, floating sub-heading word, keyword heading word, organism supplementary concept word, protocol supplementary concept word, rare disease supplementary concept word, unique identifier, synonyms, population supplementary concept word, anatomy supplementary concept word] |
| 28 | (physiological adj2 effects).mp. [mp=title, book title, abstract, original title, name of substance word, subject heading word, floating sub-heading word, keyword heading word, organism supplementary concept word, protocol supplementary concept word, rare disease supplementary concept word, unique identifier, synonyms, population supplementary concept word, anatomy supplementary concept word] |
| 29 | Oxygen Consumption/ or Physical Fitness/ or aerobic capacity.mp. |
| 30 | Energy Metabolism/ or metabolic energy.mp. |
| 31 | Fatigue/ or Muscle Fatigue/ or Mental Fatigue/ or fatigue.mp. |
| 32 | (heat adj3 exhaustion).mp. |
| 33 | pulmonary function.mp. or Respiratory Function Tests/ |
| 34 | forced vital capacity.mp. or Vital Capacity/ |
| 35 | shortness of breath.mp. or Dyspnea/ |
| 36 | (Eye adj3 irritation).mp. [mp=title, book title, abstract, original title, name of substance word, subject heading word, floating sub-heading word, keyword heading word, organism supplementary concept word, protocol supplementary concept word, rare disease supplementary concept word, unique identifier, synonyms, population supplementary concept word, anatomy supplementary concept word] |
| 37 | dehydration.mp. [mp=title, book title, abstract, original title, name of substance word, subject heading word, floating sub-heading word, keyword heading word, organism supplementary concept word, protocol supplementary concept word, rare disease supplementary concept word, unique identifier, synonyms, population supplementary concept word, anatomy supplementary concept word] |
| 38 | burn.mp. [mp=title, book title, abstract, original title, name of substance word, subject heading word, floating sub-heading word, keyword heading word, organism supplementary concept word, protocol supplementary concept word, rare disease supplementary concept word, unique identifier, synonyms, population supplementary concept word, anatomy supplementary concept word] |
| 39 | (skin adj3 inflammation).mp. [mp=title, book title, abstract, original title, name of substance word, subject heading word, floating sub-heading word, keyword heading word, organism supplementary concept word, protocol supplementary concept word, rare disease supplementary concept word, unique identifier, synonyms, population supplementary concept word, anatomy supplementary concept word] |
| 40 | cancer*.mp. |
| 41 | lung function.mp. or Forced Expiratory Volume/ |
| 42 | Inflammation Mediators/ or inflammation.mp. or Inflammation/ or Neurogenic Inflammation/ |
| 43 | bronchitis/ or pneumonia/ or respiratory distress/ |
| 44 | smoking.mp. [mp=title, book title, abstract, original title, name of substance word, subject heading word, floating sub-heading word, keyword heading word, organism supplementary concept word, protocol supplementary concept word, rare disease supplementary concept word, unique identifier, synonyms, population supplementary concept word, anatomy supplementary concept word] |
| 45 | alcohol.mp. [mp=title, book title, abstract, original title, name of substance word, subject heading word, floating sub-heading word, keyword heading word, organism supplementary concept word, protocol supplementary concept word, rare disease supplementary concept word, unique identifier, synonyms, population supplementary concept word, anatomy supplementary concept word] |
| 46 | thermal stress.mp. [mp=title, book title, abstract, original title, name of substance word, subject heading word, floating sub-heading word, keyword heading word, organism supplementary concept word, protocol supplementary concept word, rare disease supplementary concept word, unique identifier, synonyms, population supplementary concept word, anatomy supplementary concept word] |
| 47 | arrythmia.mp. [mp=title, book title, abstract, original title, name of substance word, subject heading word, floating sub-heading word, keyword heading word, organism supplementary concept word, protocol supplementary concept word, rare disease supplementary concept word, unique identifier, synonyms, population supplementary concept word, anatomy supplementary concept word] |
| 48 | hypertension.mp. [mp=title, book title, abstract, original title, name of substance word, subject heading word, floating sub-heading word, keyword heading word, organism supplementary concept word, protocol supplementary concept word, rare disease supplementary concept word, unique identifier, synonyms, population supplementary concept word, anatomy supplementary concept word] |
| 49 | lipid metabolism/ or dyslipidemia.mp. [mp=title, book title, abstract, original title, name of substance word, subject heading word, floating sub-heading word, keyword heading word, organism supplementary concept word, protocol supplementary concept word, rare disease supplementary concept word, unique identifier, synonyms, population supplementary concept word, anatomy supplementary concept word] |
| 50 | arterial stiffness.mp. [mp=title, book title, abstract, original title, name of substance word, subject heading word, floating sub-heading word, keyword heading word, organism supplementary concept word, protocol supplementary concept word, rare disease supplementary concept word, unique identifier, synonyms, population supplementary concept word, anatomy supplementary concept word] |
| 51 | airway.mp. [mp=title, book title, abstract, original title, name of substance word, subject heading word, floating sub-heading word, keyword heading word, organism supplementary concept word, protocol supplementary concept word, rare disease supplementary concept word, unique identifier, synonyms, population supplementary concept word, anatomy supplementary concept word] |
| 52 | stroke.mp. [mp=title, book title, abstract, original title, name of substance word, subject heading word, floating sub-heading word, keyword heading word, organism supplementary concept word, protocol supplementary concept word, rare disease supplementary concept word, unique identifier, synonyms, population supplementary concept word, anatomy supplementary concept word] |
| 53 | cardiac arrest.mp. [mp=title, book title, abstract, original title, name of substance word, subject heading word, floating sub-heading word, keyword heading word, organism supplementary concept word, protocol supplementary concept word, rare disease supplementary concept word, unique identifier, synonyms, population supplementary concept word, anatomy supplementary concept word] |
| 54 | musculo-skeletal/ or musculoskeletal.mp. [mp=title, book title, abstract, original title, name of substance word, subject heading word, floating sub-heading word, keyword heading word, organism supplementary concept word, protocol supplementary concept word, rare disease supplementary concept word, unique identifier, synonyms, population supplementary concept word, anatomy supplementary concept word] |
| 55 | health services.mp. [mp=title, book title, abstract, original title, name of substance word, subject heading word, floating sub-heading word, keyword heading word, organism supplementary concept word, protocol supplementary concept word, rare disease supplementary concept word, unique identifier, synonyms, population supplementary concept word, anatomy supplementary concept word] |
| 56 | (casualt* or dead or died or death* or disease* or illness* or morbidity or mortality or (health adj3 effect*) or (adverse* adj3 effect*) or (negative* adj3 impact*) or (health adj3 impact*) or (health adj3 affect*) or (negative* adj3 affect*) or (adverse* adj3 affect*) or (health adj3 problem*) or (human* adj3 health) or (health adj3 hazard*) or toxic*).ti,ab,kw,kf. |
| 57 | Hospitalization/ or Emergency Service, Hospital/ or Death/ or mortality/ or cause of death/ or child mortality/ or fatal outcome/ or fetal mortality/ or hospital mortality/ or infant mortality/ or maternal mortality/ or mortality, premature/ or perinatal mortality/ or survival rate.mp. [mp=title, book title, abstract, original title, name of substance word, subject heading word, floating sub-heading word, keyword heading word, organism supplementary concept word, protocol supplementary concept word, rare disease supplementary concept word, unique identifier, synonyms, population supplementary concept word, anatomy supplementary concept word] |
| 58 | (hospitalization or hospitalisation or ((hospital* or emergency) adj3 (visit* or admission)) or survival or casualt* or dead or died or death* or mortality).ti,kw,kf. or (hospitalization or hospitalisation or ((hospital* or emergency) adj3 (visit* or admission)) or survival or casualt* or dead or died or death* or mortality).ab. /freq=2 |
| 59 | (wounds and injuries).mp. [mp=title, book title, abstract, original title, name of substance word, subject heading word, floating sub-heading word, keyword heading word, organism supplementary concept word, protocol supplementary concept word, rare disease supplementary concept word, unique identifier, synonyms, population supplementary concept word, anatomy supplementary concept word] |
| 60 | (wound* or injury or injuries or burn or burns or smoke inhal*).ti,ab,kw,kf. |
| 61 | exp Pregnancy Complications/ or Fetal Growth Retardation/ or exp Neurodevelopmental Disorders/ or Maternal Health/ or Maternal-Fetal Exchange/ or Maternal Exposure/ or Prenatal Care/ or Pregnancy/ or Maternal Mortality/ or Maternal Welfare/ or maternal-child nursing/ or neonatal nursing/ or exp fetal monitoring/ or placental function tests/ or preimplantation diagnosis/ or exp prenatal diagnosis/ or uterine monitoring/ or Fetoscopy/ |
| 62 | (pregnan* or fetus or fetal or foetal growth or retard* or maternal or prenatal or neonatal or placenta* or uterus or uterine or fetoscop*).ti,ab,kw,kf. |
| 63 | Asthma, Occupational/ or Occupational Exposure/ or Air Pollutants, Occupational/ or occupational injuries/ |
| 64 | respiratory tract diseases/ or diagnostic techniques, respiratory system/ or Respiration/ |
| 65 | (asthma* or bronchi* or lung* or dyspnea or laboured breath* or breathing difficult* or ARDS or (respiratory adj2 (distress or disease* or illness or insufficien*))).ti,ab,kw,kf. |
| 66 | hypersensitivity/ |
| 67 | (hypersensitivity* or allerg*).ti,ab,kw,kf. |
| 68 | Eye Diseases/ or ophthalmological/ or Vision, Ocular.mp. [mp=title, book title, abstract, original title, name of substance word, subject heading word, floating sub-heading word, keyword heading word, organism supplementary concept word, protocol supplementary concept word, rare disease supplementary concept word, unique identifier, synonyms, population supplementary concept word, anatomy supplementary concept word] |
| 69 | (eye or ocular or vision or ophthalmolog*).ti,ab,kw,kf. |
| 70 | cardiovascular diseases/ or cardiovascular/ |
| 71 | (cardio* or cardiac* or heart or myocardial or pulmonary).ti,ab,kw,kf. |
| 72 | mental disorders/ or mental health/ or Behavioral Symptoms/ |
| 73 | (mental or psych* or behavio*).ti,ab,kw,kf. |
| 74 | oxidative stress.mp. [mp=title, book title, abstract, original title, name of substance word, subject heading word, floating sub-heading word, keyword heading word, organism supplementary concept word, protocol supplementary concept word, rare disease supplementary concept word, unique identifier, synonyms, population supplementary concept word, anatomy supplementary concept word] |
| 75 | cognitive.mp. [mp=title, book title, abstract, original title, name of substance word, subject heading word, floating sub-heading word, keyword heading word, organism supplementary concept word, protocol supplementary concept word, rare disease supplementary concept word, unique identifier, synonyms, population supplementary concept word, anatomy supplementary concept word] |
| 76 | hearing.mp. [mp=title, book title, abstract, original title, name of substance word, subject heading word, floating sub-heading word, keyword heading word, organism supplementary concept word, protocol supplementary concept word, rare disease supplementary concept word, unique identifier, synonyms, population supplementary concept word, anatomy supplementary concept word] |
| 77 | nutrition.mp. [mp=title, book title, abstract, original title, name of substance word, subject heading word, floating sub-heading word, keyword heading word, organism supplementary concept word, protocol supplementary concept word, rare disease supplementary concept word, unique identifier, synonyms, population supplementary concept word, anatomy supplementary concept word] |
| 78 | quality of life.mp. [mp=title, book title, abstract, original title, name of substance word, subject heading word, floating sub-heading word, keyword heading word, organism supplementary concept word, protocol supplementary concept word, rare disease supplementary concept word, unique identifier, synonyms, population supplementary concept word, anatomy supplementary concept word] |
| 79 | depression.mp. [mp=title, book title, abstract, original title, name of substance word, subject heading word, floating sub-heading word, keyword heading word, organism supplementary concept word, protocol supplementary concept word, rare disease supplementary concept word, unique identifier, synonyms, population supplementary concept word, anatomy supplementary concept word] |
| 80 | suicide.mp. [mp=title, book title, abstract, original title, name of substance word, subject heading word, floating sub-heading word, keyword heading word, organism supplementary concept word, protocol supplementary concept word, rare disease supplementary concept word, unique identifier, synonyms, population supplementary concept word, anatomy supplementary concept word] |
| 81 | anxiety.mp. [mp=title, book title, abstract, original title, name of substance word, subject heading word, floating sub-heading word, keyword heading word, organism supplementary concept word, protocol supplementary concept word, rare disease supplementary concept word, unique identifier, synonyms, population supplementary concept word, anatomy supplementary concept word] |
| 82 | lung cancer.mp. [mp=title, book title, abstract, original title, name of substance word, subject heading word, floating sub-heading word, keyword heading word, organism supplementary concept word, protocol supplementary concept word, rare disease supplementary concept word, unique identifier, synonyms, population supplementary concept word, anatomy supplementary concept word] |
| 83 | neurological.mp. [mp=title, book title, abstract, original title, name of substance word, subject heading word, floating sub-heading word, keyword heading word, organism supplementary concept word, protocol supplementary concept word, rare disease supplementary concept word, unique identifier, synonyms, population supplementary concept word, anatomy supplementary concept word] |
| 84 | drug abuse/ or drug misuse.mp. [mp=title, book title, abstract, original title, name of substance word, subject heading word, floating sub-heading word, keyword heading word, organism supplementary concept word, protocol supplementary concept word, rare disease supplementary concept word, unique identifier, synonyms, population supplementary concept word, anatomy supplementary concept word] |
| 85 | forced vital capacity/ or vital capacity/ or lung capacity.mp. [mp=title, book title, abstract, original title, name of substance word, subject heading word, floating sub-heading word, keyword heading word, organism supplementary concept word, protocol supplementary concept word, rare disease supplementary concept word, unique identifier, synonyms, population supplementary concept word, anatomy supplementary concept word] |
| 86 | leukemia/ or lymphoma/ or myeloma.mp. [mp=title, book title, abstract, original title, name of substance word, subject heading word, floating sub-heading word, keyword heading word, organism supplementary concept word, protocol supplementary concept word, rare disease supplementary concept word, unique identifier, synonyms, population supplementary concept word, anatomy supplementary concept word] |
| 87 | infection.mp. [mp=title, book title, abstract, original title, name of substance word, subject heading word, floating sub-heading word, keyword heading word, organism supplementary concept word, protocol supplementary concept word, rare disease supplementary concept word, unique identifier, synonyms, population supplementary concept word, anatomy supplementary concept word] |
| 88 | 8 or 9 or 10 or 11 or 12 or 13 or 14 or 15 or 16 or 17 or 18 or 19 or 20 or 21 or 22 or 23 or 24 or 25 or 26 or 27 or 28 or 29 or 30 or 31 or 32 or 33 or 34 or 35 or 36 or 37 or 38 or 39 or 40 or 41 or 42 or 43 or 44 or 45 or 46 or 47 or 48 or 49 or 50 or 51 or 52 or 53 or 54 or 55 or 56 or 57 or 58 or 59 or 60 or 61 or 62 or 63 or 64 or 65 or 66 or 67 or 68 or 69 or 70 or 71 or 72 or 73 or 74 or 75 or 76 or 77 or 78 or 79 or 80 or 81 or 82 or 83 or 84 or 85 or 86 or 87 |
| 89 | 7 and 88 |
| 90 | exp animals/ not humans.sh. |
| 91 | 89 not 90 |
| **No** | **Embase** |
| 1 | exp Fires/ or bushfire.mp. |
| 2 | Wildfires/ |
| 3 | Fires/ or exp Wildfires/ or Wildfire*.mp. |
| 4 | (bushfire* or bush fire* or wildfire* or wild* fire* or forest fire* or wildland fire* or wild land fire* or woodland fire* or wood land fire* or brushfire* or brush fire* or rural fire* or grassfire* or grass fire or vegetation fire* or landscape fire*).mp. [mp=title, abstract, heading word, drug trade name, original title, device manufacturer, drug manufacturer, device trade name, keyword heading word, floating subheading word, candidate term word] |
| 5 | Fires/ and (bush* or biomass or forest* or grass* or habitat* or vegetation or wild* or ecosystem* or savanna* or agricultur* or prescribed burn* or prescribed fire*).ti,ab,kw,kf. |
| 6 | (bushfire* or forestfire* or grassfire* or wildfire* or smoke pollution event* or (fire* adj3 (bush* or biomass or forest* or grass* or habitat* or vegetation or wild* or ecosystem* or savanna* or agricultur*)) or prescribed burn* or prescribed fire*).ti,ab,kw,kf. |
| 7 | 1 or 2 or 3 or 4 or 5 or 6 |
| 8 | morbidity/ or mortality/ or "cause of death"/ |
| 9 | patient care/ or hospitalization/ or patient admission/ or patient discharge/ or patient readmission/ |
| 10 | patients/ or inpatients/ |
| 11 | exp disease/ or exp disease attributes/ |
| 12 | emergency service, hospital/ or trauma centers/ |
| 13 | exp Emergency Medical Services/ |
| 14 | Ambulances/ or Air Ambulances/ |
| 15 | exp "Wounds and Injuries"/ |
| 16 | exp "diseases (non mesh)"/ or exp respiratory tract diseases/ or bronchial diseases/ or exp asthma/ or exp lung diseases/ or exp respiration disorders/ |
| 17 | exp Sleep Wake Disorders/ |
| 18 | mental health/ or mental disorders/ or exp anxiety disorders/ or exp mood disorders/ or exp "trauma and stressor related disorders"/ |
| 19 | mental health services/ or psychological problem/ or psychiatric problem*/ or occupational stress*/ or psychological distress/ or post-traumatic stress disorder/ or PTSD/ or mental fatigue/ or Burnout/ or Emotional exhaustion/ or Sleep disturbance*/ or Substance abuse/ or Job-related stressor*/ or Traumatic exposure*/ or Cumulative stress/ |
| 20 | exp pregnancy/ or exp pregnancy outcome/ |
| 21 | exp pregnancy complications/ or exp cardiovascular diseases/ |
| 22 | (health* or disease* or mortalit* or morbidit* or incidence* or hospital* or admission* or injur* or traum* or emergency or emergencies or ambulanc*).mp. |
| 23 | (asthm* or cardio* or allerg* or respirator* or COPD or lung diseas* or lung function* or mental health* or sleep* or disorder* or insomni*).mp. |
| 24 | (pregnan* or gestation* or maternal or preterm* or pre term* or pre matur* or prematur* or post matur* or postmatur* or abortion* or stillbirth* or still birth*).mp. |
| 25 | neoplasms/ or neoplasms, post-traumatic/ or eye diseases/ or cardiovascular diseases/ or immune system diseases/ or occupational diseases/ or "wounds and injuries"/ |
| 26 | (physiological adj3 response*).mp. |
| 27 | (Physiological adj3 well-being).mp. [mp=title, abstract, heading word, drug trade name, original title, device manufacturer, drug manufacturer, device trade name, keyword heading word, floating subheading word, candidate term word] |
| 28 | (physiological adj2 effects).mp. [mp=title, abstract, heading word, drug trade name, original title, device manufacturer, drug manufacturer, device trade name, keyword heading word, floating subheading word, candidate term word] |
| 29 | Oxygen Consumption/ or Physical Fitness/ or aerobic capacity.mp. |
| 30 | Energy Metabolism/ or metabolic energy.mp. |
| 31 | Fatigue/ or Muscle Fatigue/ or Mental Fatigue/ or fatigue.mp. |
| 32 | (heat adj3 exhaustion).mp. |
| 33 | pulmonary function.mp. or Respiratory Function Tests/ |
| 34 | forced vital capacity.mp. or Vital Capacity/ |
| 35 | shortness of breath.mp. or Dyspnea/ |
| 36 | (Eye adj3 irritation).mp. [mp=title, abstract, heading word, drug trade name, original title, device manufacturer, drug manufacturer, device trade name, keyword heading word, floating subheading word, candidate term word] |
| 37 | dehydration.mp. [mp=title, abstract, heading word, drug trade name, original title, device manufacturer, drug manufacturer, device trade name, keyword heading word, floating subheading word, candidate term word] |
| 38 | burn.mp. [mp=title, abstract, heading word, drug trade name, original title, device manufacturer, drug manufacturer, device trade name, keyword heading word, floating subheading word, candidate term word] |
| 39 | (skin adj3 inflammation).mp. [mp=title, abstract, heading word, drug trade name, original title, device manufacturer, drug manufacturer, device trade name, keyword heading word, floating subheading word, candidate term word] |
| 40 | cancer*.mp. |
| 41 | lung function.mp. or Forced Expiratory Volume/ |
| 42 | Inflammation Mediators/ or inflammation.mp. or Inflammation/ or Neurogenic Inflammation/ |
| 43 | bronchitis/ or pneumonia/ or respiratory distress/ |
| 44 | smoking.mp. [mp=title, abstract, heading word, drug trade name, original title, device manufacturer, drug manufacturer, device trade name, keyword heading word, floating subheading word, candidate term word] |
| 45 | alcohol.mp. [mp=title, abstract, heading word, drug trade name, original title, device manufacturer, drug manufacturer, device trade name, keyword heading word, floating subheading word, candidate term word] |
| 46 | thermal stress.mp. [mp=title, abstract, heading word, drug trade name, original title, device manufacturer, drug manufacturer, device trade name, keyword heading word, floating subheading word, candidate term word] |
| 47 | arrythmia.mp. [mp=title, abstract, heading word, drug trade name, original title, device manufacturer, drug manufacturer, device trade name, keyword heading word, floating subheading word, candidate term word] |
| 48 | hypertension.mp. [mp=title, abstract, heading word, drug trade name, original title, device manufacturer, drug manufacturer, device trade name, keyword heading word, floating subheading word, candidate term word] |
| 49 | lipid metabolism/ or dyslipidemia.mp. [mp=title, abstract, heading word, drug trade name, original title, device manufacturer, drug manufacturer, device trade name, keyword heading word, floating subheading word, candidate term word] |
| 50 | arterial stiffness.mp. [mp=title, abstract, heading word, drug trade name, original title, device manufacturer, drug manufacturer, device trade name, keyword heading word, floating subheading word, candidate term word] |
| 51 | airway.mp. [mp=title, abstract, heading word, drug trade name, original title, device manufacturer, drug manufacturer, device trade name, keyword heading word, floating subheading word, candidate term word] |
| 52 | stroke.mp. [mp=title, abstract, heading word, drug trade name, original title, device manufacturer, drug manufacturer, device trade name, keyword heading word, floating subheading word, candidate term word] |
| 53 | cardiac arrest.mp. [mp=title, abstract, heading word, drug trade name, original title, device manufacturer, drug manufacturer, device trade name, keyword heading word, floating subheading word, candidate term word] |
| 54 | musculo-skeletal/ or musculoskeletal.mp. [mp=title, abstract, heading word, drug trade name, original title, device manufacturer, drug manufacturer, device trade name, keyword heading word, floating subheading word, candidate term word] |
| 55 | health services.mp. [mp=title, abstract, heading word, drug trade name, original title, device manufacturer, drug manufacturer, device trade name, keyword heading word, floating subheading word, candidate term word] |
| 56 | (casualt* or dead or died or death* or disease* or illness* or morbidity or mortality or (health adj3 effect*) or (adverse* adj3 effect*) or (negative* adj3 impact*) or (health adj3 impact*) or (health adj3 affect*) or (negative* adj3 affect*) or (adverse* adj3 affect*) or (health adj3 problem*) or (human* adj3 health) or (health adj3 hazard*) or toxic*).ti,ab,kw,kf. |
| 57 | Hospitalization/ or Emergency Service, Hospital/ or Death/ or mortality/ or cause of death/ or child mortality/ or fatal outcome/ or fetal mortality/ or hospital mortality/ or infant mortality/ or maternal mortality/ or mortality, premature/ or perinatal mortality/ or survival rate.mp. [mp=title, abstract, heading word, drug trade name, original title, device manufacturer, drug manufacturer, device trade name, keyword heading word, floating subheading word, candidate term word] |
| 58 | (hospitalization or hospitalisation or ((hospital* or emergency) adj3 (visit* or admission)) or survival or casualt* or dead or died or death* or mortality).ti,kw,kf. or (hospitalization or hospitalisation or ((hospital* or emergency) adj3 (visit* or admission)) or survival or casualt* or dead or died or death* or mortality).ab. /freq=2 |
| 59 | (wounds and injuries).mp. [mp=title, abstract, heading word, drug trade name, original title, device manufacturer, drug manufacturer, device trade name, keyword heading word, floating subheading word, candidate term word] |
| 60 | (wound* or injury or injuries or burn or burns or smoke inhal*).ti,ab,kw,kf. |
| 61 | exp Pregnancy Complications/ or Fetal Growth Retardation/ or exp Neurodevelopmental Disorders/ or Maternal Health/ or Maternal-Fetal Exchange/ or Maternal Exposure/ or Prenatal Care/ or Pregnancy/ or Maternal Mortality/ or Maternal Welfare/ or maternal-child nursing/ or neonatal nursing/ or exp fetal monitoring/ or placental function tests/ or preimplantation diagnosis/ or exp prenatal diagnosis/ or uterine monitoring/ or Fetoscopy/ |
| 62 | (pregnan* or fetus or fetal or foetal growth or retard* or maternal or prenatal or neonatal or placenta* or uterus or uterine or fetoscop*).ti,ab,kw,kf. |
| 63 | Asthma, Occupational/ or Occupational Exposure/ or Air Pollutants, Occupational/ or occupational injuries/ |
| 64 | respiratory tract diseases/ or diagnostic techniques, respiratory system/ or Respiration/ |
| 65 | (asthma* or bronchi* or lung* or dyspnea or laboured breath* or breathing difficult* or ARDS or (respiratory adj2 (distress or disease* or illness or insufficien*))).ti,ab,kw,kf. |
| 66 | hypersensitivity/ |
| 67 | (hypersensitivity* or allerg*).ti,ab,kw,kf. |
| 68 | Eye Diseases/ or ophthalmological/ or Vision, Ocular.mp. [mp=title, abstract, heading word, drug trade name, original title, device manufacturer, drug manufacturer, device trade name, keyword heading word, floating subheading word, candidate term word] |
| 69 | (eye or ocular or vision or ophthalmolog*).ti,ab,kw,kf. |
| 70 | cardiovascular diseases/ or cardiovascular/ |
| 71 | (cardio* or cardiac* or heart or myocardial or pulmonary).ti,ab,kw,kf. |
| 72 | mental disorders/ or mental health/ or Behavioral Symptoms/ |
| 73 | (mental or psych* or behavio*).ti,ab,kw,kf. |
| 74 | oxidative stress.mp. [mp=title, abstract, heading word, drug trade name, original title, device manufacturer, drug manufacturer, device trade name, keyword heading word, floating subheading word, candidate term word] |
| 75 | cognitive.mp. [mp=title, abstract, heading word, drug trade name, original title, device manufacturer, drug manufacturer, device trade name, keyword heading word, floating subheading word, candidate term word] |
| 76 | hearing.mp. [mp=title, abstract, heading word, drug trade name, original title, device manufacturer, drug manufacturer, device trade name, keyword heading word, floating subheading word, candidate term word] |
| 77 | nutrition.mp. [mp=title, abstract, heading word, drug trade name, original title, device manufacturer, drug manufacturer, device trade name, keyword heading word, floating subheading word, candidate term word] |
| 78 | quality of life.mp. [mp=title, abstract, heading word, drug trade name, original title, device manufacturer, drug manufacturer, device trade name, keyword heading word, floating subheading word, candidate term word] |
| 79 | depression.mp. [mp=title, abstract, heading word, drug trade name, original title, device manufacturer, drug manufacturer, device trade name, keyword heading word, floating subheading word, candidate term word] |
| 80 | suicide.mp. [mp=title, abstract, heading word, drug trade name, original title, device manufacturer, drug manufacturer, device trade name, keyword heading word, floating subheading word, candidate term word] |
| 81 | anxiety.mp. [mp=title, abstract, heading word, drug trade name, original title, device manufacturer, drug manufacturer, device trade name, keyword heading word, floating subheading word, candidate term word] |
| 82 | lung cancer.mp. [mp=title, abstract, heading word, drug trade name, original title, device manufacturer, drug manufacturer, device trade name, keyword heading word, floating subheading word, candidate term word] |
| 83 | neurological.mp. [mp=title, abstract, heading word, drug trade name, original title, device manufacturer, drug manufacturer, device trade name, keyword heading word, floating subheading word, candidate term word] |
| 84 | drug abuse/ or drug misuse.mp. [mp=title, abstract, heading word, drug trade name, original title, device manufacturer, drug manufacturer, device trade name, keyword heading word, floating subheading word, candidate term word] |
| 85 | forced vital capacity/ or vital capacity/ or lung capacity.mp. [mp=title, abstract, heading word, drug trade name, original title, device manufacturer, drug manufacturer, device trade name, keyword heading word, floating subheading word, candidate term word] |
| 86 | leukemia/ or lymphoma/ or myeloma.mp. [mp=title, abstract, heading word, drug trade name, original title, device manufacturer, drug manufacturer, device trade name, keyword heading word, floating subheading word, candidate term word] |
| 87 | infection.mp. [mp=title, abstract, heading word, drug trade name, original title, device manufacturer, drug manufacturer, device trade name, keyword heading word, floating subheading word, candidate term word] |
| 88 | 8 or 9 or 10 or 11 or 12 or 13 or 14 or 15 or 16 or 17 or 18 or 19 or 20 or 21 or 22 or 23 or 24 or 25 or 26 or 27 or 28 or 29 or 30 or 31 or 32 or 33 or 34 or 35 or 36 or 37 or 38 or 39 or 40 or 41 or 42 or 43 or 44 or 45 or 46 or 47 or 48 or 49 or 50 or 51 or 52 or 53 or 54 or 55 or 56 or 57 or 58 or 59 or 60 or 61 or 62 or 63 or 64 or 65 or 66 or 67 or 68 or 69 or 70 or 71 or 72 or 73 or 74 or 75 or 76 or 77 or 78 or 79 or 80 or 81 or 82 or 83 or 84 or 85 or 86 or 87 |
| 89 | 7 and 88 |
| 90 | exp animals/ not humans.sh. |
| 91 | 89 not 90 |
| **Scopus** | |
| (TITLE-ABS-KEY ( "bushfire*" OR "bush fire*" OR "wildfire*" OR "wild* fire*" OR "forest fire*" OR "wildland fire*" OR "wild land fire*" OR "woodland fire*" OR "wood land fire*" OR "brushfire*" OR "brush fire*" OR "rural fire*" OR "grassfire*" OR "grass fire*" OR "vegetation fire*" OR "landscape fire*" ) AND TITLE-ABS-KEY ("health*" OR "disease*" OR "mortalit*" OR "death*" OR "morbidit*" OR "incidence*" OR "hospital*" OR "admission*" OR "injur*" OR "traum*" OR "emergency*" OR "emergencies" OR "ambulanc*" OR "asthm*" OR "allerg*" OR "cadio*" OR "respirator*" OR "copd" OR "lung diseas*" OR "lung function*" OR "mental health*" OR "sleep*" OR "disorder*" OR "insomni*" OR "pregnan*" OR "gestation*" OR "maternal" OR "preterm*" OR "preterm" OR "prematur*" OR "postmatur*" OR "abortion*" OR "stillbirth*" OR "still birth*" ) AND NOT TITLE-ABS-KEY ( "animal*" OR "not human*" )) | |
